# The role of small extracellular vesicle-miRNAs in endometriosis

**DOI:** 10.1093/humrep/dead216

**Published:** 2023-10-24

**Authors:** Hannah M Nazri, Erin Greaves, Siobhan Quenby, Rebecca Dragovic, Thomas T Tapmeier, Christian M Becker

**Affiliations:** Division of Biomedical Sciences, Warwick Medical School, University of Warwick, Coventry, UK; Division of Biomedical Sciences, Warwick Medical School, University of Warwick, Coventry, UK; Division of Biomedical Sciences, Warwick Medical School, University of Warwick, Coventry, UK; Nuffield Department of Women’s & Reproductive Health, Endometriosis CaRe Centre, University of Oxford, Oxford, UK; Nuffield Department of Women’s & Reproductive Health, Endometriosis CaRe Centre, University of Oxford, Oxford, UK; Department of Obstetrics and Gynaecology, Monash University, Clayton, VIC, Australia; The Ritchie Centre, Hudson Institute of Medical Research, Clayton, VIC, Australia; Nuffield Department of Women’s & Reproductive Health, Endometriosis CaRe Centre, University of Oxford, Oxford, UK

**Keywords:** exosomes, small extracellular vesicles, exosomal microRNAs, miRNAs, sEV-miRNAs, endometriosis

## Abstract

Endometriosis is defined by the presence of extrauterine endometrial-like tissue, which can cause pain and infertility in 10% of reproductive-age women. To date, the pathogenesis is poorly understood resulting in significant diagnostic delays and poor therapeutic outcomes in many women. Small extracellular vesicles (sEVs) (<200 nm) are cell-derived vesicles containing molecules that can influence gene expression and behaviour in target cells. One such cargo are microRNAs (miRNAs), which are short, non-coding RNAs mostly 19–25 nucleotides in length that regulate post-transcriptional gene expression. This mini-review focuses on the role of sEV-miRNAs, which are conceivably better biomarkers for endometriosis than free miRNAs, which reflect the true pathophysiological state in the body, as sEV-encapsulated miRNAs are protected from degradation compared to free miRNA and provide direct cell-to-cell communication via sEV surface proteins. sEV-miRNAs have been implicated in the immunomodulation of macrophages, the proliferation, migration and invasion of endometrial cells, and angiogenesis, all hallmarks of endometriosis. The diagnostic potential of sEV-miRNA was investigated in one study that reported the sensitivity and specificity of two sEV-miRNAs (hsa-miR-22-3p and hsa-miR-320a-3p) in distinguishing endometriosis from non-endometriosis cases. Only three studies have explored the therapeutic potential of sEV-miRNAs *in vivo* in mice—two looked into the role of sEV-hsa-miR-214-3p in decreasing fibrosis, and one investigated sEV-hsa-miR-30c-5p in suppressing the invasive and migratory potential of endometriotic lesions. While early results are encouraging, studies need to further address the potential influence of factors such as the menstrual cycle as well as the location and extent of endometriotic lesions on miRNA expression in sEVs. Given these findings, and extrapolating from other conditions such as cancer, diabetes, and pre-eclampsia, sEV-miRNAs could present an attractive and urgently needed future diagnostic and therapeutic target for millions of women suffering from endometriosis. However, research in this area is hampered by lack of adherence to the International Society for Extracellular Vesicles 2018 guideline in separating and characterising sEVs, as well as the World Endometriosis Research Foundation Endometriosis Phenome and Biobanking Harmonisation Project protocols.

## Graphical abstract

**Graphical Abstract:**
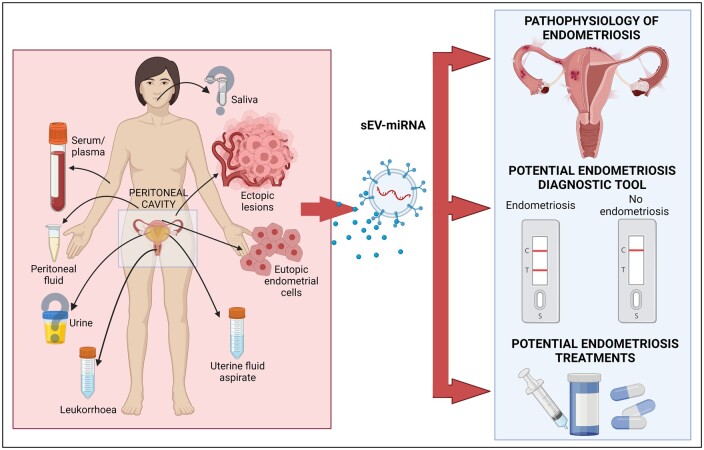
**sEV-miRNAs, which can be isolated from all biological fluids, are essential in the pathophysiology of endometriosis, and potentially in the diagnosis and treatment of endometriosis.** This figure was created with BioRender.com. sEV-miRNA, small extracellular vesicle-microRNA.

## Introduction

Endometriosis, the presence of extrauterine endometrial-like lesions, affects ∼5–10% of reproductive-aged females (Zondervan *et al.*, 2018) although rare premenarcheal manifestations and postmenopausal endometriosis have been described (Gemmell *et al.*, 2017). Globally, an estimated 190 million reproductive-aged women suffer from endometriosis (Zondervan *et al.*, 2020) although the true prevalence might be higher.

Women with endometriosis often experience severe menstrual and non-menstrual pain involving the lower abdomen, pelvis or lumbosacral region, deep dyspareunia, dyschezia, and dysuria (Uimari *et al.*, 2021) and infertility in 30–50% of cases (Prescott *et al.*, 2016). Patients with endometriosis suffer from chronic fatigue (Ramin-Wright *et al.*, 2018) and are at a higher risk of psychiatric disturbances (56.4%) than those without endometriosis (43.6%) (Pope *et al.*, 2015). Endometriosis is further associated with poorer health-related quality of life mean scores and work productivity as compared to asymptomatic controls (Nnoaham et al., 2011) resulting in an estimated €9.9 billion (equivalent to £8.4 billion) in societal cost in 2012 (Simoens *et al.*, 2012)—a higher economic burden should be assumed today.

Endometriotic lesions most commonly present as superficial peritoneal/serosal lesions of different colours and sizes, as ovarian endometriotic cysts or mostly fibrotic (deep) nodules >5 mm below the peritoneal surface (Cornillie *et al.*, 1990). The extent of pelvic endometriosis is widely described using the revised American Society for Reproductive Medicine (rASRM) classification system into stages I–IV based on direct observation of lesions during surgery (Canis *et al.*, 1997). Infrequently, extrapelvic locations, including abdominal surgical scars (Wang *et al.*, 2016), the umbilicus, diaphragm, thorax, pericardium, and lymph nodes (Ceccaroni *et al.*, 2012), have been described.

To date, the cause of endometriosis is not fully defined. Sampson’s theory of retrograde menstruation (Sampson, 1927), does not explain why only some women develop endometriosis as retrograde menstruation is physiological (Halme *et al.*, 1984). Insufficient public and professional awareness, and the trivilisation of women’s pain (Shah *et al.*, 2010; Samulowitz *et al.*, 2018; De Sanctis *et al.*, 2020), absence of clinically relevant biomarkers (May *et al.*, 2010; Liu *et al.*, 2015; Gupta *et al.*, 2016; Nisenblat *et al.*, 2016a,b,c), and the unspecific nature of endometriosis-associated symptoms (Zondervan *et al.*, 2018) are some of the challenges in diagnosing endometriosis. Previously, the gold standard for diagnosing endometriosis was diagnostic laparoscopy, which may have prevented earlier diagnosis of endometriosis. The latest ESHRE guideline on endometriosis no longer recommends diagnostic laparoscopy as the gold standard diagnostic tool, reserving it rather for women with negative MRI or ultrasound imaging, and/or where empirical treatment has failed (Becker *et al.*, 2022). Current endometriosis treatments are mainly symptomatic and rarely curative while also aiming for fertility preservation if required. All these factors contribute to the 6–8 years (average 6.7 years, measured in 16 clinical centres across 10 countries) waiting time between symptoms onset and endometriosis diagnosis (Nnoaham et al., 2011).

### Extracellular vesicles

**Figure 1.**
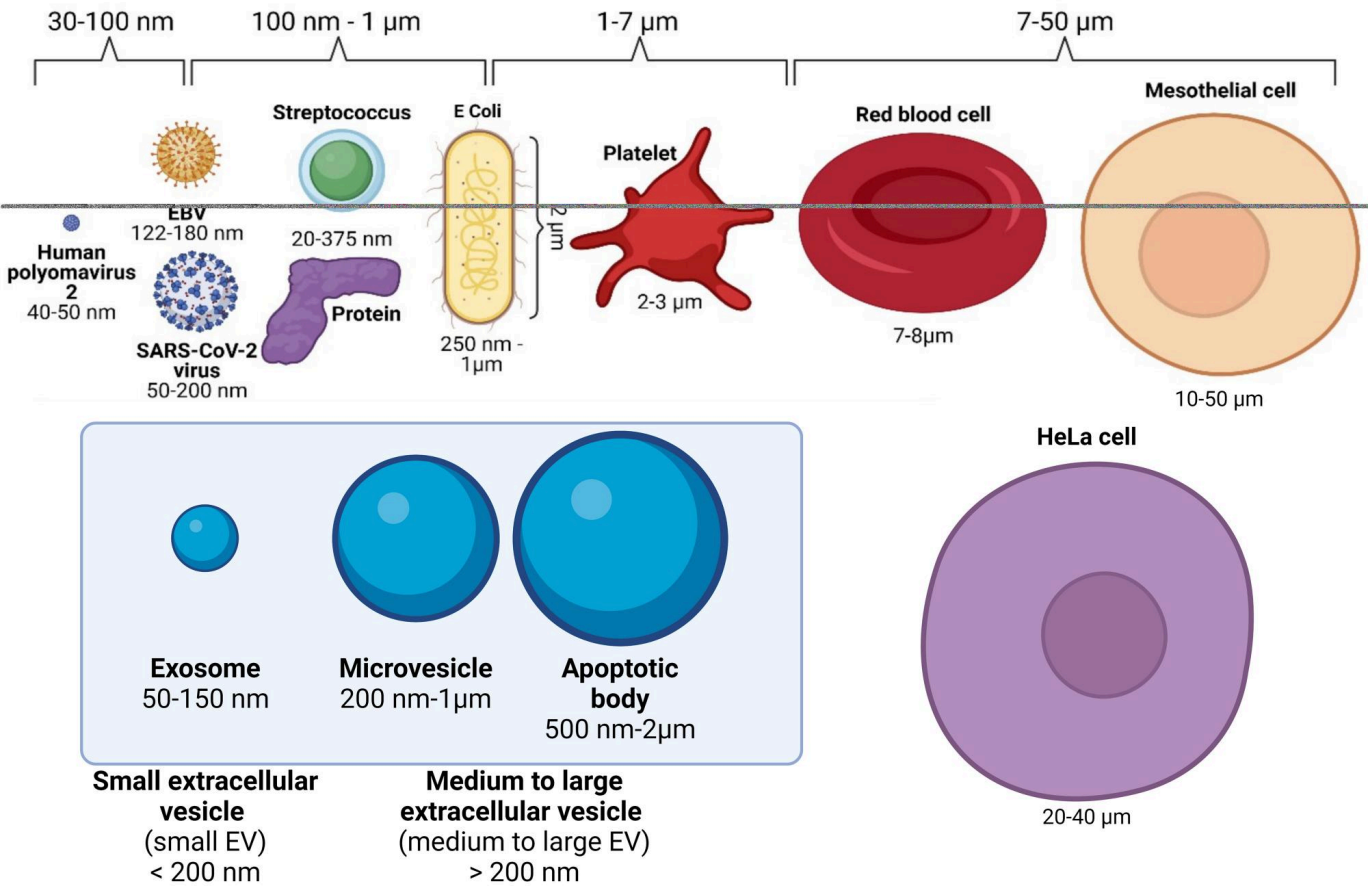
**The diversity of extracellular vesicles produced by cells in the human body.** Compared to exosomes, MVs can be defined as EVs that are released directly from the plasma membrane and are on an average larger than exosomes, although their sizes could range from 30 nm to 1 µm (Jeppesen *et al.*, 2019). The terms ‘ectosomes’ and ‘microparticles’ are synonymously used to describe MVs. Like exosomes, MVs are essential for cellular communication and carry cargo such as mRNA, miRNA, lncRNA, and protein. Apoptotic bodies are released as cells undergo apoptotic cell disassembly, where the plasma membrane blebs and the apoptotic membrane protrudes and fragments. Therefore, different EV subtypes can be categorised according to their biogenesis, size, constituent protein, and isolation methods; however, the different criteria for EV subtypes often overlap, and can even contradict each other. This figure was created with BioRender.com. EV, extracellular vesicle; MV, microvesicle; mRNA, messenger RNA; miRNA, microRNA; lncRNA, long non-coding RNA.

Extracellular vesicles (EV) are nano-sized membrane-bound vesicles produced by almost all cells in the body. EVs comprise a heterogeneous population and over the years various terms have been used to describe these vesicles, including exosomes, ectosomes, microvesicles (MVs), and microparticles (Fig. 1). The first investigations to describe EVs were two studies (Harding *et al.*, 1984; Pan *et al.*, 1985) that observed 50 nm vesicles released from maturing reticulocytes with transferrin receptors through the process of intraluminal budding of multivesicular endosomes that bind to the lipid cell membrane before their release into the circulation. These vesicles were named ‘exosomes’ by Rose Johnstone, although before the 1980s the term had been used to describe different phenomena such as ‘membrane fragments’ (De Broe *et al.*, 1977), platelet ‘dust’, or cellular debris (Wolf, 1967). Ostensibly, the blood-derived pro-coagulant membrane-derived particles isolated by Chargaff and West (1946) were the platelet ‘dust’ isolated by Wolf (1967), therefore it could be argued that EVs were first observed in 1946.The term was even used to describe isolated 40–1000 nm ‘exosome complexes’ secreted by neoplastic cell lines with a 5′ nucleotidase activity (Trams *et al.*, 1981). Nevertheless, in 1996, these EVs gained renewed research interest with the exosome secretion of Epstein–Barr-virus-transformed B cells (Raposo *et al.*, 1996).

Exosomes are small EVs [sEVs, defined as <200 nm (Théry *et al.*, 2018)] of 30–150 nm in size. They are initially formed as an early endosome (EE) through the inward budding of the plasma membrane as it recycles receptors, proteins, and lipids back into the cell. As the EE matures into a late endosome (LE), it traffics and catalogues proteins from the Golgi apparatus, as well as genetic material from the cytoplasm, for degradation or transportation outside of the cell. The LE then forms the multivesicular body (MVB) with the invagination of the MVB membrane to form intraluminal vesicles (ILVs). When ILVs are released into the extracellular space via fusion of the MVB with the cell plasma membrane, they are defined as exosomes. Owing to their biogenesis, exosomes contain proteins involved in the MVB formation (e.g. ALIX, and TSG101), membrane trafficking (e.g. Rab GTPases and annexins), and are enriched in tetraspanins (e.g. CD9, CD63, and CD81) (van Niel *et al.*, 2006; Zöller, 2009; Raposo and Stoorvogel, 2013).

However, there is no consensus on specific markers or specific separation methods for delineating different EV subtypes, beyond their size (Fig. 1). There is no one separation method superior to the other, with one method sacrificing yield versus purity, and vice versa (Théry *et al.*, 2018; Jia *et al.*, 2022). The isolation method of choice will thus depend on the goal of the study, the amount and quality of sample biofluids, and the downstream analyses chosen. The studies included in this mini-review used differential ultracentrifugation (low purity containing protein complexes, medium yield, time consuming, suitable for large sample numbers), size exclusion chromatography (medium-to-high yield, high purity, suitable for low sample numbers), and ready-made isolation kits with precipitation buffers (high yield, low purity as contain polymers, suitable for large sample numbers) or magnetic capture (may be susceptible to charged particles in the sample) (Xu *et al.*, 2016; Jia *et al.*, 2022). The International Society for Extracellular Vesicles (ISEV) 2018 recommendations state that researchers should use the generic term EV unless the precise biogenesis can be captured using live imaging techniques (Théry *et al.*, 2018). ISEV also recommends further describing EV subtypes based on physical and biochemical characteristics and/or conditions/sources (Théry *et al.*, 2018). In this review, we utilise the term sEV, even though many of the studies we include may originally have referred to ‘exosomes’.

sEVs are detected in a variety of bodily fluids (van Niel *et al.*, 2022). They transport cargo that can affect gene expression of target cells in remote parts of the body. Introduction of new receptors, proteins, or genetic material through sEV fusion with the target cells confer new cellular properties that mirror the parent cell’s expression (Zomer *et al.*, 2015). sEVs are implicated in various disease pathologies, including cancer (Hoshino *et al.*, 2015; Zomer *et al.*, 2015), diabetes (Noren Hooten and Evans, 2020), cardiovascular disease (Liu *et al.*, 2021), neurodegenerative disease (Vandendriessche *et al.*, 2020) as well as in benign obstetric and gynaecological conditions such as pre-eclampsia (Murugesan *et al.*, 2022) and endometriosis (Harp *et al.*, 2016; Nazri *et al.*, 2020). A urinary sEV RNA-based diagnostic tool for prostate cancer (Kretschmer *et al.*, 2022), the ExoDx™ Prostate (IntelliScore) (EPI) test, has been used to aid clinical decisions for >50 000 patients so far and is included in the United States National Comprehensive Cancer Network guidelines for early prostate cancer detection.

sEV cargos include the RNA species such as mRNA, piwi-interacting RNA, transfer RNA fragments, microRNA (miRNA), long non-coding RNA (lncRNA), and rRNA (O’Brien *et al.*, 2020). miRNAs are short, non-coding RNAs of ∼19–25 nucleotides in length, which are synthesised via DNA transcription into primary miRNAs, then processed in the cytoplasm into precursor miRNAs, and mature miRNAs (O’Brien et al., 2018). miRNAs regulate post-transcriptional gene expression mainly via targeting mRNAs at the 3′ untranslated region to inhibit mRNA translation into protein or enhance mRNA instability, leading to its degradation (O’Brien et al., 2018). miRNAs can also upregulate gene expression in certain microribonucleoprotein (miRNP) factors or cellular conditions (Vasudevan, 2012) by controlling mRNA transcription rate and translation into proteins as they are shuttled between different subcellular compartments (Makarova *et al.*, 2016).

While the role of miRNA as a diagnostic and therapeutic tool for endometriosis in itself has been an area of burgeoning research interest (Agrawal *et al.*, 2018), sEV-miRNA is better protected from degradation than free miRNA and thus conceivably the better biomarker candidate as it arguably better reflects the true pathophysiological state in the body (Cheng *et al.*, 2014).

In this mini-review, we have included all possible original articles that investigated sEV miRNAs in endometriosis regardless of sEV separation or characterisation methods (Table 1). Literature searches were carried out on PubMed using the terms ‘endometriosis small extracellular vesicles miRna’, ‘endometriosis exosomes miRna’, and ‘endometriosis exosomes miRna’ and studies selected according to quality of hypothesis and methodological rigour in exploring the hypothesis. Review articles were excluded from this mini-review.

**Table 1:** Studies investigating the differential expression of sEV-miRNA.

No.	Sample type	Sample size	sEV isolation methods	sEV concentration and size	sEV protein markers	Methods	Dysregulated sEV miRNA	Study
1	Serum	Human samples: Endometriosis (stages I–IV, diagnosed via laparoscopy and confirmed with histology), n=25 Control (tubal factor infertility), n=25 Mentioned about unbalanced menstrual cycle phases in the discussion but did not declare number of samples that were of proliferative, secretory, or menstrual phases.	Differential ultracentrifugation	NTA: Mean concentration: 3.7 × 10^8^ particles/ml. Mean size: 93±4.1 nm. Visualised via TEM.	WB: CD9 and CD63 positive.	Microarray analysis: Endometriosis, n=5 Control, n=5 followed by qRT-PCR: Endometriosis, n=20 Control, n=20	↑ hsa-miR-22-3p, hsa-miR-320a-3p	Zhang *et al.* (2020a)

2	Serum	Human samples: Endometriosis (stages I–II, n=12; III–IV, n=30, diagnosed via laparoscopy), n=42 *Proliferative phase samples, n=16, secretory phase samples, n=20, presumably unknown, n=6 Control (benign ovarian teratoma, n = 17; simple ovarian cyst n = 7), n=24 *Proliferative phase samples, n=14, secretory phase samples, n=11, total numbers do not match	MagCapture™ Exosome Isolation Kit PS (FUJIFILM Wako, Japan)	TEM: 60–80 nm. NTA not done.	WB: CD9 and CD63 positive.	Microarray analysis: Endometriosis (stage I, n=1; stage III, n=2; stage IV, n=1), n=4 Control, n=4 followed by qRT-PCR: Endometriosis, n=42 Control, n=24	↑ hsa-miR-26b-5p, hsa-miR-215-5p ↓ hsa-miR-6795-3p	Wu *et al.* (2022)

3	Serum Eutopic endometrium Endometriotic lesion Endometriosis mouse model	Human samples: Endometriosis (ovarian endometrioma, from ovarian cystectomy or oopherectomy and confirmed with histology) and control (hysterectomy for other pelvic masses): *Serum, n=20 each Eutopic endometrium, n=unclear Endometriotic lesion, n=unclear *sEV source **Proliferative phase samples Samples from mice: Endometriosis mouse model, n=24, divided into 4 treatment groups	ExoQuick-TC Exosome Isolation Kit (System Biosciences SBI, USA)	TEM: 90–120 nm. NTA not done.	WB: CD9 and CD63 positive.	Immunohistochemistry and WB for CCN2, α-SMA, and collagen α1. miRNA extraction of all cell types and qRT-PCR for hsa-miR-214-3p. Transfection of all cell types with hsa-miR-214-3p/mimics/NC and qRT-PCR for miRNA and CCN2. Co-culture of transfected and untransfected cells followed by qRT-PCR for miRNA and CCN2. Serum sEV-miRNA: qRT-PCR. Endometriosis mouse model: sEV-miRNA uptake experiment, qRT-PCR.	↓ hsa-miR-214-3p	Zhang *et al.* (2021)
4	Plasma Eutopic endometrium Endometriotic lesion PF	Human samples: Endometriosis (stages III–IV, diagnosed via laparoscopy): Plasma, n=6 Eutopic endometrium, n=6 Endometriotic lesion, n=6 PF, n=6 Control (free from endometriosis, infertility, and gynaecological malignancies): No other information given *Matched, secretory phase samples	MiRCURY Exosome Isolation Kit (Qiagen)	TEM: 30–150 nm. NTA not done.	WB: CD63 positive, calnexin negative.	sEV-miRNA sequencing of all sample types followed by qRT-PCR. Proteomics experiments (out of review scope). sEV uptake experiments by endometriotic lesion/eutopic endometrium-endothelial co-culture.	↑ hsa-miR-27a-3p ↓ hsa-miR-30d-5p, hsa-miR-375 Regardless of sample types	Khalaj *et al.* (2019)

5	Eutopic endometrium Endometriotic lesion	Human samples: Endometriosis (diagnosed via laparoscopy): eutopic endometrium, n=5; endometriotic lesion, n=5 Control (n=5, with 4 patients with subserosal fibroids <3 cm): Eutopic endometrium, n=5 *Secretory phase samples	Total Exosome Isolation kit (Invitrogen)	NTA: Size: 20–200 nm with peak size at 35 nm. Visualised via TEM.	Not done.	sEV uptake experiment by HUVECs. qRT-PCR for hsa-miR-21-5p and hsa-miR-126-5p of miRNA from sEVs and cells followed by sequencing of total miRNA.	↑ hsa-miR-21-5p Between endometriotic lesion and eutopic endometrium	Harp *et al.* (2016)

6	Eutopic endometrium	Human samples: Endometriosis (stages II–IV, infertile, diagnosed via laparoscopy), n=3 Control (fertile, post-operative CIN II–III, malignancy excluded), n=3 *Secretory phase samples **CIN: cervical intraepithelial neoplasia	ExoQuick-TC Exosome Isolation Kit (System Biosciences SBI, USA)	NTA: Size: 30–150 nm. Visualised via TEM.	Flow cytometry: CD63 and CD81 positive.	sEV-miRNA sequencing followed by qRT-PCR.	↑ 26 miRNAs ↓ 23 miRNAs	Zhou *et al.* (2020)

7	Eutopic endometrium Endometrioma	Human samples: Endometriosis (stages III–IV): Endometrioma, n=13 Eutopic endometrium, n=13 Control (tubal factor infertility), n=13 *Proliferative phase samples	ExoQuick-TC Exosome Isolation Kit (System Biosciences SBI, USA)	NTA done and sEVs visualised via TEM according to ‘Materials & Methods’ section but results not reported in the study.	WB done according to ‘Materials & Methods’ section but results not reported in the study.	sEV-miRNA sequencing: Endometrioma, eutopic endometrium, control endometrium, n=3 each. qRT-PCR: Endometrioma, eutopic endometrium, control endometrium, n=10 each. Dual-luciferase reporter gene assay.	↓ hsa-miR-15a-5p	Wu *et al.* (2021)
8	Eutopic endometrium Endometriotic lesion Endometriosis mouse model	Human samples: Endometriosis (stage III–IV, ovarian endometrioma): Paired eutopic endometrium and *endometriotic lesion, n=24 *sEV source Control (laparoscopic myomectomy): Eutopic endometrium, n=8 Samples from mice: Endometriosis mouse model, n=12 divided into 3 treatment groups **No information about cycle phases	Ultracentrifugation	TEM: 50–100 nm. Dynamic light scattering analysis and zeta potential analysis: ∼65 nm particles (−20.1 mV).	Not done.	Cell culture for hsa-miR-214-3p transfection: Paired eutopic endometrium and endometriotic lesion, n=10, followed by miRNA extraction and qRT-PCR for hsa-miR-214-3p and GAPDH and mRNA extraction and qRT-PCR for CTGF, collagen αI and GAPDH detection. WB for CTGF, αSMA, collagen αI: Control eutopic endometrium, n=2 Endometriotic lesion, n=2 *In situ* hybridisation with hsa-miR-214-3p probes: Endometriotic lesion, n=6 Control eutopic endometrium, n=6 followed by qRT-PCR. Immunohistochemistry for CTGF, αSMA, collagen αI of all human cell types. Endometriosis mouse model: sEV-miRNA uptake experiments, qRT-PCR and histology.	↓ hsa-miR-214-3p	Wu *et al.* (2018)

9	Eutopic endometrium Endometriotic lesion Endometriosis mouse model	Human samples: Endometriosis (stage III–IV, ovarian endometrioma, diagnosed via laparoscopy, middle to late proliferative phase): Eutopic endometrium, *endometriotic lesion, n=24 *sEV source Control (tubal factor infertility): *Eutopic endometrium, n=20 *sEV source Samples from mice: Endometriosis mouse model, n=24 divided into 2 treatment groups *Middle to late stage of proliferative phase	Differential ultracentrifugation, followed by exosome precipitation using a reagent	NTA: ∼ 80 nm. TEM: 50–100 nm.	WB: CD9, CD63, and HSP70 positive.	Cell culture and transfection of hsa-miR-30c-5p/mimic/inhibitor±BCL9 and corresponding NC. miRNA sequencing and microarray analysis of eutopic and ectopic endometrial tissues: Endometriosis, n=5 Control, n=5 qRT-PCR of miRNA isolated from sEVs, tissues, and cells: Endometriotic lesion, n=24 Eutopic endometrium, n=20 - Unclear number of sEV samples used sEV co-culture experiments followed by qRT-PCR. sEV transwell and wound healing assay. Dual-luciferase reporter gene assay. Endometriosis mouse model: sEV uptake experiments followed by qRT-PCR, immunohistochemistry.	↓ hsa-miR-30c-5p	Zhang *et al.* (2022)
10	Endometriotic lesion Serum	Human samples: *Endometriotic lesion from endometriosis patients, n=unclear *Normal human serum (no further information) *sEV source **No information about cycle phases	Differential ultracentrifugation	NTA: Endometriosis sEV, 83.68±16.85 nm Normal human serum sEV, 79.64±17.33 nm Visualised via TEM.	WB: CD63 and TSG101 positive. Calnexin negative.	sEV treatment of macrophages, followed by flow cytometry for pro-repair (M2) marker and WB for PTEN and PI3K. Transfection of macrophages with hsa-miR-301a-3p/mimic/negative control and then sEV treatment followed by qRT-PCR, flow cytometry for pro-repair (M2) marker, and WB for Arg-1, PTEN, and PI3K.	↑ hsa-miR-301a-3p	Huang *et al.* (2022)

11	PF	Human samples: Endometriosis (stages I, n=17; II, n=11; III, n=15; IV, n=11, diagnosed via laparoscopy), n=54 Control (ovarian dermoid cyst, n=9; uterine leiomyoma, n=4, diagnosed via laparoscopy), n=13 *Proliferative phase samples	Differential ultracentrifugation	No information in study.	No information in study.	sEV-miRNA sequencing: Early endometriosis, n=3 Advanced endometriosis, n=3 Control, n=3 followed by qRT-PCR.	↓ hsa-miR-130-5p	Chen *et al.* (2019)

12	PF (peritoneal macrophages) Endometriotic lesions	Human samples: Endometriosis (diagnosed via laparoscopy and confirmed with histology): PF, n=20 Endometriotic lesions, n=unclear Control (uterine leiomyoma, tubal ligation): PF, n=20 *sEV from peritoneal macrophages **No information about cycle phases	Differential ultracentrifugation	NTA: Mean concentration: 4.1 × 10^8^ particles/ml. Mean size: 105±3.9 nm. Visualised via TEM.	WB: CD9 and CD63 positive.	Cell migration and invasion assays of sEV-treated endometriotic lesion. Microarray analysis of sEV-miRNA followed by qRT-PCR. Transfection of endometriotic lesions with hsa-miR-22-3p mimic/inhibitor/corresponding NC, followed by qRT-PCR. Dual-luciferase reporter gene assay.	↑ hsa-miR-22-3p	Zhang *et al.* (2020b)
13	Uterine aspirate fluid	Human samples: Endometriosis (stage III–IV, endometrioma, diagnosed via laparoscopy), n=22 Control (including simple ovarian cyst, uterine leiomyoma, tubal ligation), n=25 *Mentioned about that findings were regardless of secretory or proliferative phases in the results section but did not declare number of samples that were of proliferative or secretory.	Size exclusion chromatography exosome isolation kit (Echobiotech, Beijing, China)	NTA: Median size: 111.7 nm and the proportion of the main peak was 94.1%. Visualised via TEM.	WB: CD63, TSG101, and HSP70 positive. Calnexin negative.	Microarray analysis of miRNA from sEVs and endometrial tissues from uterine aspirate fluid, followed by qRT-PCR. sEV uptake experiments with JNK activator anisomycin by M0 macrophages followed by flow cytometry, WB, and MAPK phosphorylation antibody array. Transwell migration and invasion assay: Co-culture of endometrial tissues of control group and sEV-treated macrophages. Transfection of M0 macrophages with hsa-miR-210-3p mimic/inhibitor/corresponding NC followed by qRT-PCR and flow cytometry.	↑ hsa-miR-210-3p Regardless of secretory or proliferative phases	Jiang *et al.* (2022)

14	Eutopic endometrium Leukorrhoea	Human samples: Endometriosis, n=11 Control (hysteroscopic submucosal myomectomy, n=6; normal physical examinations, n=5), n=11 *No information about cycle phases	Differential ultracentrifugation	NTA: 60–150 nm, with mean size of 95.5 nm. Visualised via TEM.	WB: CD63 and HSP70 positive.	Microarray analysis of sEV-miRNA followed by qRT-PCR: Endometriosis, n=6 Control, n=5	↑ hsa-miR-202-3p, hsa-miR-202-5p	Zheng *et al.* (2023)

sEV-miRNA, small extracellular vesicle-microRNA; miRNA, microRNA; NTA, nanoparticle tracking analyses; TEM, transmission electron microscopy; WB, Western blot; qRT-PCR, quantitative real time PCR; PF, peritoneal fluid; TSG101, tumour susceptibility gene 101; HSP70, 70 kilodalton heat shock protein; CTGF, connective tissue growth factor—also known as CCN2; α-SMA, α-smooth muscle actin; NC, negative control; HUVEC, human umbilical vein endothelial cells; BCL9, B-cell lymphoma 9; PTEN, phosphatase and tensin homolog; PI3K, phosphatidylinositol 3-kinase; MAPK, mitogen-activated protein kinase.

## sEV-miRNA and endometriosis pathophysiology

**Figure 2.**
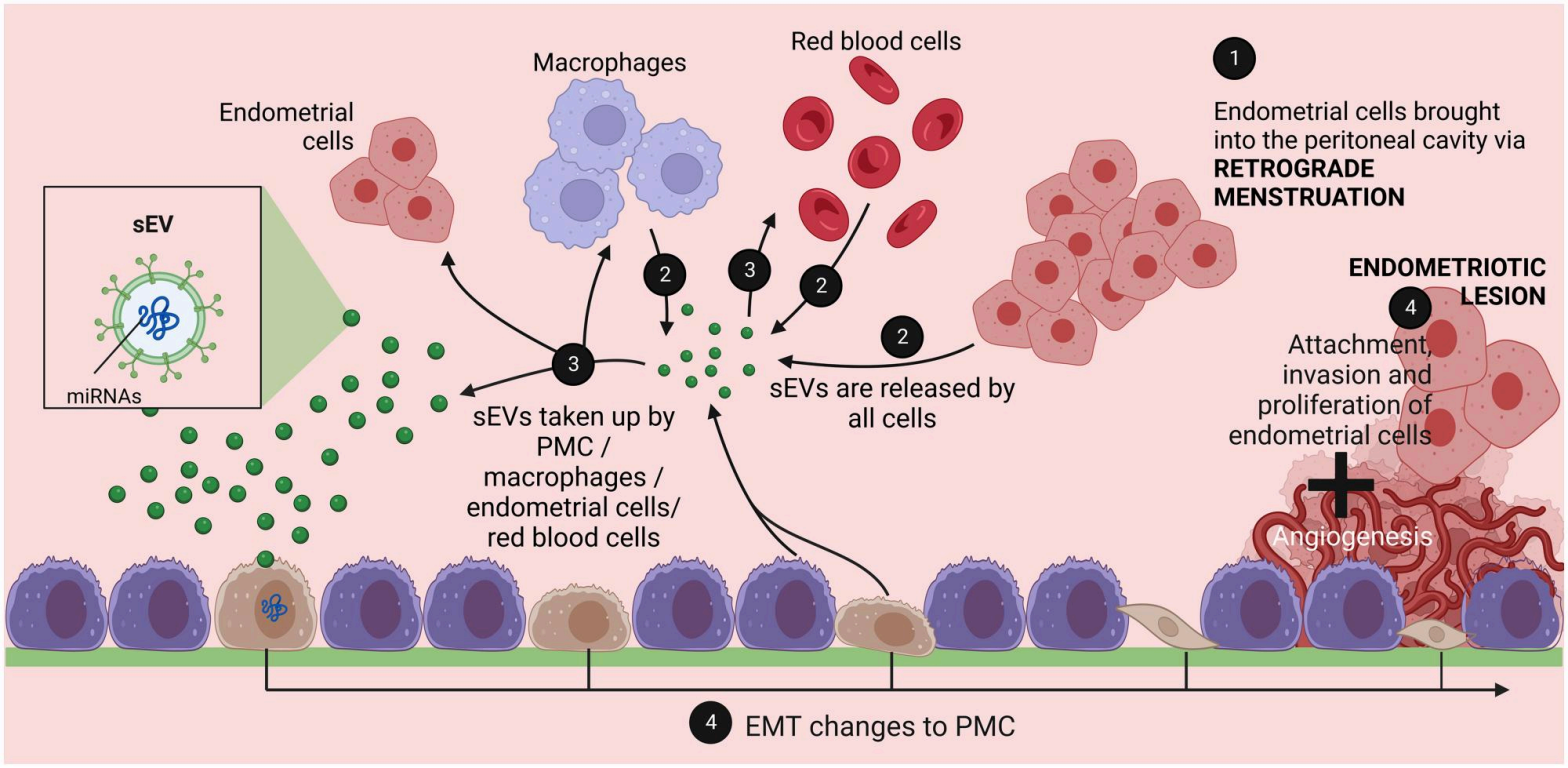
**Retrograde menstruation is only one part of the endometriosis pathophysiology.** (1) Retrograde menstruation brings endometrial cells into the peritoneal cavity. (2) sEVs are produced by all cells (endometrial cells, red blood cells, pMφ, andPMCs) in the peritoneal cavity. (3) sEVs containing miRNAs are taken up by endometrial cells, red blood cells, pMφ, and PMC, causing changes to recipient cells. (4) Uptake of sEVs and internalisation of miRNAs promotes proliferation, migration, and invasion of endometrial cells and of existing ectopic lesions, immunomodulation of macrophages, and potentially EMT changes of PMCs, although none of the studies in this review investigated the impact of sEV-miRNA on PMCs. PF-derived sEVs are likely to originate from a variety of cell types including endometrial cells, red blood cells, immune cells, ectopic lesions, and PMCs. This figure was created with BioRender.com. sEV, small extracellular vesicle; miRNA, microRNA; pMφ, peritoneal macrophage; PMC, peritoneal mesothelial cell; EMT, epithelial-to-mesenchymal transition; PF, peritoneal fluid; sEV-miRNA, small extracellular vesicle-microRNA.

**Figure 3.**
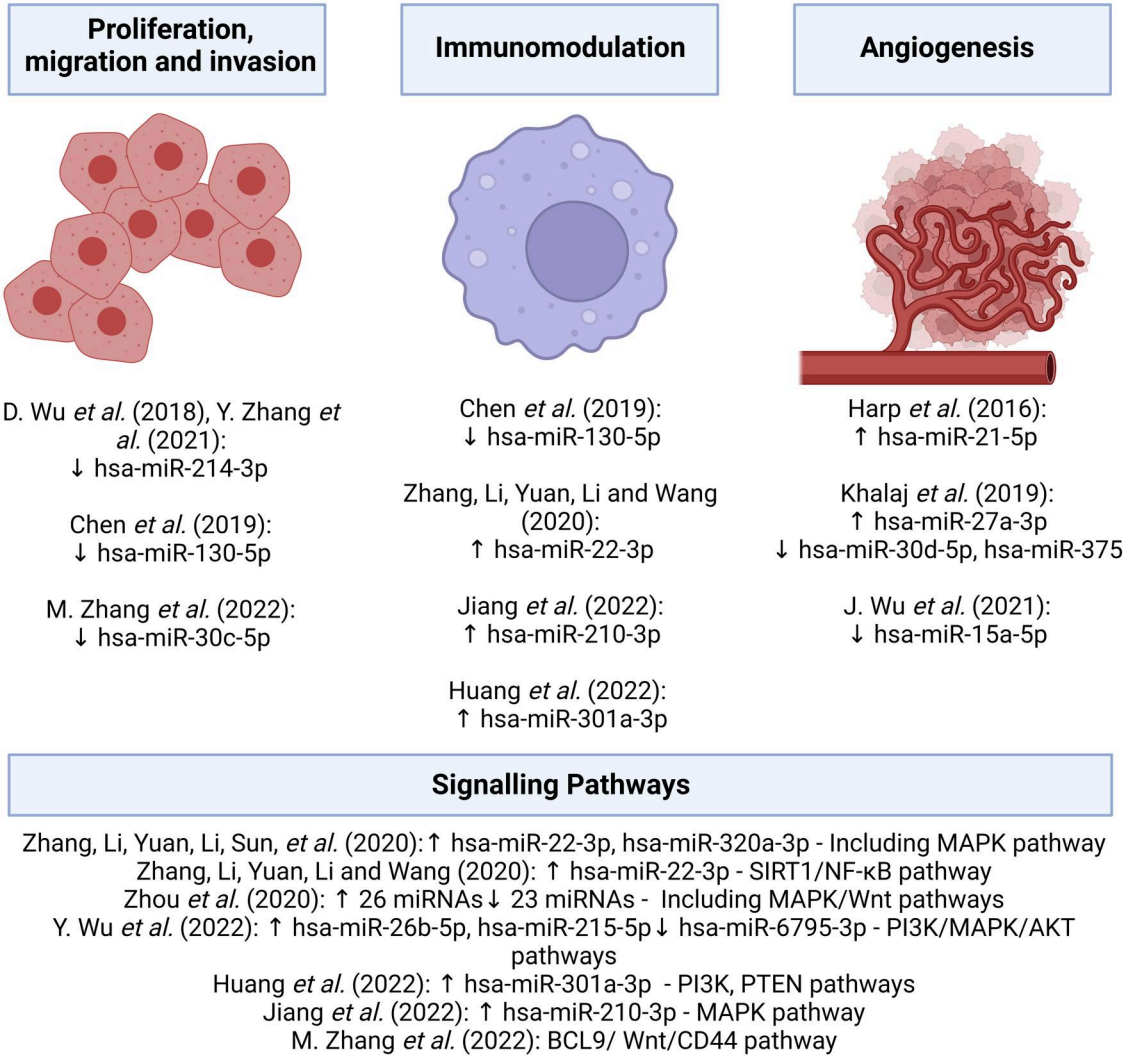
**Proposed sEV-miRNAs involved in the pathophysiology of endometriosis.** sEV-miRNAs involved in the pathophysiology of endometriosis through the proliferation, migration, and invasive potential of endometrial cells, immunomodulation, and angiogenesis (formation of blood vessels) to support explanted endometrial cells (ectopic lesions). Studies also investigated endometriosis-specific miRNAs and the signalling pathways involved. This figure was created with BioRender.com. sEV-miRNA, small extracellular vesicle-microRNA.

The roles of sEV-miRNA have been proposed in several key mechanisms of endometriosis pathophysiology (Table 1, Figs 2 and 3), for instance in the immunomodulation of peritoneal macrophages (pMφ), proliferation and migration of ectopic endometriosis lesions (Zhang *et al.*, 2020b, 2022; Huang *et al.*, 2022), and angiogenesis (Harp *et al.*, 2016; Khalaj *et al.*, 2019). Differential expression of sEV-miRNAs isolated from serum/plasma (Khalaj *et al.*, 2019; Zhang *et al.*, 2020a, 2021; Wu *et al.*, 2022), leukorrhoea (Zheng *et al.*, 2023), eutopic endometrium (Khalaj *et al.*, 2019; Zhou *et al.*, 2020; Wu *et al.*, 2021; Zheng *et al.*, 2023), endometrioma (Wu *et al.*, 2021), endometriotic lesion (Harp *et al.*, 2016; Wu *et al.*, 2018; Zhang *et al.*, 2022), peritoneal fluid (PF) (Chen *et al.*, 2019; Khalaj *et al.*, 2019; Zhang *et al.*, 2020b), and uterine aspirate (Jiang *et al.*, 2022) has been demonstrated in sEVs isolated from women with endometriosis compared to controls.

### Immunomodulation, proliferation, and migration

Retrogradely spilt menstrual blood, cells, and tissue can be detected in the abdominal cavity of most women at the time of menstruation (Halme *et al.*, 1984). Therefore, an impaired immune response in combination with the increased proliferative and migratory activity of these cells may contribute to the establishment of endometriosis in some women (Zondervan *et al.*, 2018).

Serum-derived sEVs from women with endometriosis contained higher hsa-miR-22-3p and hsa-miR-320a-3p levels compared to controls, with elevated hsa-miR-22-3p levels in stages III–IV versus stages I–II endometriosis (Zhang *et al.*, 2020a). High hsa-miR-22-3p and hsa-miR-320a-3p levels were also correlated with high serum CA-125 levels in patients with endometriosis (Zhang *et al.*, 2020a). Kyoto Encyclopaedia of Genes and Genomes (KEGG) analysis suggested mitogen-activated protein kinase (MAPK) activity, among others (Zhang *et al.*, 2020a). Increased pMφ-derived sEV-hsa-miR-22-3p in patients compared to controls regulates the sirtuin 1/nuclear factor κB (SIRT1/NF-κB) signalling pathway leading to enhanced ectopic endometrial stromal cells (ESCs) proliferation, migration, and invasion (Zhang *et al.*, 2020b).

Pro-repair phenotype polarisation and reduced phagocytic activity in Mφ exposed to lesion-derived sEVs were mediated by hsa-miR-301a-3p through phosphatidylinositol 3-kinase (PI3K) upregulation and tumour suppressor phosphatase and tensin homologue deleted on chromosome 10 (PTEN) downregulation (Huang *et al.*, 2022). These findings echoed a previous study in an endometriosis mouse model which also revealed decreased total weight and volume of endometriotic lesions following treatment with ESC-derived sEVs (Sun *et al.*, 2019b). Elevated hsa-miR-210-3p in uterine-aspirate-fluid-derived sEVs and eutopic endometrium of patients with endometriosis decreased CD80^+^ Mφ (a surface marker of pro-inflammatory Mφ) by suppressing c-Jun N-terminal kinase (JNK, protein kinase in MAPK pathway) phosphorylation in Mφ, thus increasing pro-repair Mφ, which could escape immune surveillance. In support of these findings, sEV-treated Mφ increased proliferation, migration, and invasion of endometriosis ESCs compared to control ESCs in a transwell experiment (Jiang *et al.*, 2022). In another study, quantitative RT-PCR-verified endometriosis-specific serum-derived sEV-hsa-miR-26b-5p (downregulated), hsa-miR-215-5p (downregulated), and hsa-miR-6795-3p (upregulated) were found to be involved in the PI3K, MAPK, and protein kinase B (Akt) pathways in a KEGG analysis. Serum-derived sEV-hsa-miR-26b-5p expression was lower in rASRM stages III–IV versus stages I–II endometriosis (Wu *et al.*, 2022).

In a KEGG analysis of 49 differentially expressed sEV-miRNAs from eutopic endometrium of women with endometriosis-related infertility and control endometrium, the Wnt signalling pathway (Zhou *et al.*, 2020) was also implicated, in addition to MAPK, confirming previous findings (Wu *et al.*, 2022; Zhang *et al.*, 2022). Homeobox A10 (HOXA10) and/or leukaemia inhibitory factor (LIF)—targets of 12 miRNAs—were decreased in eutopic endometrium of women with endometriosis-related infertility compared to controls (Zhou *et al.*, 2020).

Higher levels of leukorrhoea-derived sEV-hsa-miR-202-3p and hsa-miR-202-5p in patients with endometriosis compared to controls corresponded to levels in endometriosis eutopic endometrium (Zheng *et al.*, 2023).

### Angiogenesis

Neo-angiogenesis, the formation of new blood vessels from existing ones, is a process involved in many physiological (e.g. corpus luteum formation, endometrial proliferation) and pathological processes (e.g. cancer, chronic inflammation) (Folkman, 2001). Retrogradely transplanted endometriotic cells and tissue require a blood supply for proliferation (May and Becker, 2008).

Cultured human umbilical vein endothelial cells (HUVEC) treated with eutopic ESC-derived sEVs from patients with endometriosis exhibited more extensive tube formation compared to those treated with equivalent sEVs from controls (Harp *et al.*, 2016). In this study, ectopic lesion-derived sEVs contained elevated (pro-angiogenic) hsa-miR-21-5p compared to eutopic ESC-derived sEVs from controls. However, it is uncertain what assumptions can be made from this comparison without also comparing eutopic ESC-derived and possibly PF-derived sEVs from patients and controls. Although no specific sEV-miRNAs were investigated, Sun *et al.* (2019a) observed greater tube formation in endometriosis eutopic ESC-derived sEV-treated HUVECs compared to control eutopic ESC-derived sEVs. Treating HUVECs with plasma-derived sEVs from patients with endometriosis revealed similar findings (Khalaj *et al.*, 2019). Sun *et al.* (2019a) also observed increased neurite outgrowth induced by endometriosis eutopic ESC-derived sEVs compared to control.

In an RNA sequencing study of control and endometriosis eutopic ESC- and endometrioma-derived sEV-RNA, hsa-miR-15a-5p, involved in vascular endothelial growth factor A (*VEGF-A)* downregulation, was lower in the cell culture supernatant of patients with endometriosis versus control (Wu *et al.*, 2021). The relation between hsa-circ_0026129, hsa-miR-15a-5p, and *ATP6V1A* was uncovered in an sEV-competing endogenous RNA (cERNA) network analysis. This showed that increased sEV-hsa-circ_0026129 correlated with decreased hsa-miR-15a-5p and increased *ATP6V1A* expression in ovarian endometriomas. Dual luciferase reporter assays confirmed that hsa-circ_0026129 competitively binds to hsa-miR-15a-5p, which contributed to low hsa-miR-15a-5p levels thus increasing *ATP6V1A* expression in ovarian endometriomas compared to controls. *ATP6V1A* is an oncogene of endometrial cancer, which is linked to the severity of endometriosis and endometrial receptivity (Wu *et al.*, 2021).

### PF-derived sEV-miRNAs

PF is rich in immune cells, prostaglandins, interleukin, cytokines, and growth factors that can influence the growth of endometrial lesions (Koninckx, 1998), and owing to its proximity and direct contact with endometriotic lesions, investigating PF for endometriosis-specific sEVs seems a rational approach. Studying the PF could identify the different sEV populations from different cells (e.g. pMφ, red blood cells, endometrial cells, ectopic lesions, peritoneal mesothelial cells), and correlating PF-derived sEV-miRNAs in more accessible fluids (e.g. serum, urine, or saliva) may provide a comprehensive picture of sEV-miRNAs involved in endometriosis and their utility in diagnostics.

In total, four studies investigated PF-derived sEVs in endometriosis. Out of these studies, two groups investigated PF-derived sEV-proteins (Khalaj *et al.*, 2019; Nazri *et al.*, 2020). At present, three studies investigated PF-derived sEV-miRNAs in endometriosis (Chen *et al.*, 2019; Khalaj *et al.*, 2019) including one study reporting on pMφ-derived sEVs (Zhang *et al.*, 2020b).

Increased PF-derived sEV-hsa-miR-130-3p levels, implicated in immune cell function, were found in patients with endometriosis versus controls. In stage III–IV endometriosis, increased PF-derived sEV-hsa-miR-451a and hsa-miR-486-5p were found (Chen *et al.*, 2019). Khalaj *et al.* (2019) attempted to compare the small RNA and proteome content of endometriotic lesion-derived sEVs versus patients’ matched eutopic endometrium and controls, as well as PF- and plasma-derived sEVs. Accounting for sample types, sEV-miRNAs unique to endometriosis include hsa-miR-30d-5p (upregulated), hsa-miR-27a-3p (downregulated), and hsa-miR-375 (downregulated). Using TargetScan (an online repository for miRNA biological target prediction), Khalaj *et al.* identified hsa-miR-30d-5p binding sites for thrombospondin-2, alpha-2-antiplasmin, and interferon regulatory factor 4, whereas hsa-miR-375 has binding sites for platelet-derived growth factor subunit A—all of which suggested angiogenesis and pro-inflammatory pathways (Khalaj *et al.*, 2019).

## sEV-miRNAs as diagnostic markers for endometriosis

While there is considerable potential for sEV-miRNAs to serve as diagnostic markers for endometriosis, so far only one study has reported the sensitivity and specificity of sEV-miRNAs in diagnosing endometriosis (Zhang *et al.*, 2020a). For serum-derived sEV-hsa-miR-22-3p, the AUC was higher at 85.5% than the AUC of hsa-miR-320a-3p at 82.7%. Combining both miRNAs, the AUC was 88.3% (Zhang *et al.*, 2020a), which is considered to be an encouraging AUC score in discriminating between patients with endometriosis and controls.

## sEV-miRNAs for endometriosis treatment

Fourteen studies had identified the differential expression of sEV-miRNAs in endometriosis (Table 1), however, only three studies (Wu *et al.*, 2018; Zhang *et al.*, 2021, 2022) investigated the therapeutic potential of sEV-miRNA in endometriosis *in vivo*, as described below.

### sEV-hsa-miR-214-3p

Endometriosis is a heterogeneous disease with a large range of phenotypes (Zondervan *et al.*, 2020). Although red lesions have been shown to express high levels of VEGF and other growth factors, and are therefore considered by some as the most active, fibrosis is a molecular hallmark and a frequently encountered clinical problem (Burney, 2022). Therefore, non-surgical treatment and/or prevention of fibrosis is an urgently unmet clinical need.

In a study investigating liver fibrosis (Chen *et al.*, 2014), sEV-hsa-miR-214-3p decreased the expression of connective tissue growth factor (CTGF) consequently decreasing the fibrosis of hepatic stellate cells. Thus, hsa-miR-214-3p could be a promising therapeutic target for other fibrotic diseases, including endometriosis, by targeting CTGF. In an endometriosis rodent model (induced by i.p. injection of human endometriosis eutopic ESCs), fibrosis reduction in ectopic lesions was observed when treated with human ectopic ESC-derived sEVs transfected with hsa-miR-214-3p mimics, through CTGF and collagen reduction in ESCs and endometrial epithelial cells (EECs) (n = 3), compared to mice treated with control sEVs (n = 3) and PBS (n = 3) (Wu *et al.*, 2018).

Using a modified, Burns *et al.* (2012) protocol to induce endometriosis in nude mice (n = 24)—where one experimental group was treated with uterine tissue derived from CTGF-knock-out mice and the other with uterine tissue from wild-type mice—the above findings (Wu *et al.*, 2018) were confirmed (Zhang *et al.*, 2021). The endometriosis-induced mice (n = 24) were divided into four treatment groups (n = 6) of i.p. injections of saline, human serum-derived sEVs, hsa-miR-214-3p mimics, or sEV-hsa-miR-214-3p. This revealed increased fibrosis-related protein expression in endometriotic lesions of sEV-hsa-miR-214-3p-treated mice compared to other treatment groups. Additionally, no differences were seen in mice induced with CTGF-knock-out uterine tissue, confirming the sEV-hsa-miR-214-3p’s CTGF-dependent actions. Finally, hsa-miR-214-3p downregulation and CTGF upregulation in ectopic lesions and eutopic endometrium of patients with endometriosis compared to eutopic endometrium of controls (Wu *et al.*, 2018; Zhang *et al.*, 2021) correlated with lower serum sEV-hsa-miR-214-3p in patients with endometriosis (Zhang *et al.*, 2021).

### sEV-hsa-miR-30c-5p

A hypothesis of endometriosis pathophysiology (Nazri *et al.*, 2020) had likened the formation of the endometriotic lesion to the ‘seed and soil’ hypothesis of cancer (Paget, 1889), with sEVs causing increased invasive and migratory potential of retrogradely menstruated endometrial cells, similar to how tumour metastasis is instigated by sEVs (Hoshino *et al.*, 2015). In a study by Zhang *et al.* (2022), ectopic EECs from endometrioma patients produced sEVs with low hsa-miR-30c-5p, leading to BCL9 (B-cell lymphoma 9) overexpression. BCL9, a co-activator of the Wnt/β-catenin pathway has been implicated in breast cancer metastasis (Wang et al., 2021) and hence may shed light on the invasive and migratory potential of endometriotic lesions.

In contrast, EEC-derived sEVs from control patients transferred hsa-miR-30c-5p, which targets BCL9, thus suppressing the invasive and migratory potential of ectopic EECs (Zhang *et al.*, 2022). Blockage of the BCL9/Wnt/CD44 pathway by sEV-hsa-miR-30c-5p overexpression attenuated ectopic EEC invasive potential in an endometriosis nude mice model injected with sEVs pre-treated with hsa-miR-30c-5p mimic (n = 12, each). Notably, BCL9, Wnt1, β-catenin, c-myc, cyclin D1, CD44, vimentin, and N-cadherin expression was reduced, and E-cadherin increased, corresponding to increased hsa-miR-30c-5p levels. Indeed, attenuating the BCL9/Wnt/CD44 pathway via sEV-hsa-miR-30c-5p overexpression was associated with fewer ectopic nodules in the intestinal walls of nude mice with endometriosis compared to controls (Zhang *et al.*, 2022).

## Discussion

Endometriosis is a common heterogeneous disease which lacks clinically reliable non-invasive diagnostic tools as well as disease-specific therapeutic approaches. In this review, we present the currently available literature on sEV-miRNA and its diagnostic and therapeutic potential in the disease. Their roles were studied in various biological fluids and tissues including PF, serum and plasma, leukorrhoea, uterine aspirate fluid, endometrioma as well as eutopic and ectopic endometriotic lesions. However, no studies investigated sEV-miRNAs from peritoneal mesothelial cells (Fig. 2), and since they play a central role in endometriosis (Koks *et al.*, 2000; Nair *et al.*, 2008; Griffith *et al.*, 2010; Knudtson *et al.*, 2020) this is a clear gap in the field and one that warrants further investigation. Additionally, no groups have investigated saliva- or urine-derived sEV-miRNAs.

Of the 14 studies included here, six focused on rASRM stages III–IV endometriosis (Wu *et al.*, 2018, 2021; Khalaj *et al.*, 2019; Zhang *et al.*, 2021, 2022; Jiang *et al.*, 2022). Four studies did not give information about the rASRM stages of endometriosis samples (Harp *et al.*, 2016; Zhang *et al.*, 2020b; Huang *et al.*, 2022; Zheng *et al.*, 2023). One study investigated rASRM stages II–IV (Zhou *et al.*, 2020) and only three studies investigated all rASRM stages (Chen *et al.*, 2019; Zhang *et al.*, 2020a; Wu *et al.*, 2022) although data analyses according to rASRM stage were not included in these studies. Investigating stages I–II endometriosis separately from stages III–IV is essential as increasing evidence shows that these two combined stages of endometriosis could represent different disease entities altogether, with different genetic and biological pathways underpinning the pathophysiology (Painter *et al.*, 2011; Sapkota *et al.*, 2017; Uimari *et al.*, 2017). In addition, testing sEV-miRNAs for their diagnostic potential would arguably be more relevant for superficial disease, which is difficult to visualise even using modern imaging technologies (Nisenblat *et al.*, 2016a).

An additional problem is posed by the control samples used for some studies. Samples from women suffering from other gynaecological abnormalities, such as uterine leiomyomas, ovarian cysts, and ovarian teratomas, may confound results (Table 1). For example, one study reported that sEV-miRNA from human uterine leiomyoma cells was associated with increased proliferation and angiogenesis of uterine leiomyomata (Navarro *et al.*, 2022). Similarly, sEVs from simple ovarian cysts and ovarian cancer have been used to stimulate lymphocytes to understand the differences in gene expression (Li *et al.*, 2017). Three studies had recruited patients with tubal factor infertility as controls (Zhang *et al.*, 2020a, 2022; Wu *et al.*, 2021), although the current implications of this condition on sEV populations are not known. Arguably the most suitable controls in these studies would be those with pelvic pain and negative laparoscopic findings, i.e. symptomatic controls (Tapmeier *et al.*, 2020), although the difficulty of obtaining appropriate controls is widely recognised.

None of the studies investigated sEV-miRNA in all rASRM stages of endometriosis and all phases of the menstrual cycle, including menstruation. As endometriosis is an oestrogen-dependent disease, one could speculate that the changes in sEV cargo and miRNAs may correlate to changes in oestrogen and progesterone levels. Two studies acquired samples in proliferative and secretory phases (Jiang *et al.*, 2022; Wu *et al.*, 2022), four studies only in the proliferative phase (Chen *et al.*, 2019; Wu *et al.*, 2021; Zhang *et al.*, 2021, 2022), and three studies in the secretory phase (Harp *et al.*, 2016; Khalaj *et al.*, 2019; Zhou *et al.*, 2020). Five studies provided no information about the menstrual cycle phases of their samples (Wu *et al.*, 2018; Zhang *et al.*, 2020a,b; Huang *et al.*, 2022; Zheng et al.; 2023). Therefore, regrettably, at the time of writing no research groups have yet analysed and compared sEV-miRNA and -protein in *all* menstrual cycle phases and *all* rASRM stages of patients with endometriosis and controls.

Only one study demonstrated the potential for sEV-miRNA as a diagnostic tool for endometriosis although not without the limitations discussed above (Zhang *et al.*, 2020a). Three studies (Wu *et al.*, 2018; Zhang *et al.*, 2021, 2022) evaluated the potential therapeutic properties of sEV-miRNAs *in vivo*. However, what remains to be investigated is whether treating endometriosis with sEV-has-miR-214-3p (Wu *et al.*, 2018; Zhang *et al.*, 2021) or has-miR-30c-5p (Zhang *et al.*, 2022) would translate to endometriosis symptom relief.

Most importantly, none of these studies could justify using the term ‘sEVs’ or ‘exosomes’ as defined by the ISEV 2018 guidelines (Théry *et al.*, 2018). The recommendations stipulated the use of certain separation methods for specifc downstream analyses taking into account the biological fluid the sEVs are derived from, knowing that there is a trade-off between yield and purity. Compared to ultracentrifugation, differential centrifugation or size-exclusion chromatography, isolating sEVs using precipitation methods (Harp *et al.*, 2016; Khalaj *et al.*, 2019; Zhou *et al.*, 2020; Wu *et al.*, 2021; Zhang *et al.*, 2021) results in a high yield but risks sacrificing EV purity (Gurunathan *et al.*, 2019). To be able to use the term ‘sEVs’, studies must show quantiative analyses of particle yield, with a global concentration of particle yield and particle-to-protein or particle-to-lipid ratios, as well as demonstrating EV proteins from at least three categories (transmembrane, cytosolic, purity control proteins), and visualising the sEVs as single particles. Four studies did not report any quantitative analyses of the particles (Table 1) (Chen *et al.*, 2019; Khalaj *et al.*, 2019; Zhang *et al.*, 2021; Wu *et al.*, 2022). Three studies neglected to perform experiments to confirm sEV proteins (Harp *et al.*, 2016; Wu *et al.*, 2018; Chen *et al.*, 2019). Note that Chen *et al.* (2019) did not provide any information about its EV characterisation methods and Wu *et al.* (2021) only mentioned the EV characterisation methods that they employed in their study but did not share the results. Therefore, none of the studies that performed sEV confirmation experiments satisfied the ISEV requirements. With promising results from these studies (Table 1), it is unfortunate that these studies fall short of the minimum requirements of sEV separation methods.

Any study must be independently replicated and validated in a separate cohort, ideally in large multi-national, multi-centre cohort studies. For such studies to be comparable, they should follow ISEV guidance with regards to sEV isolation (Théry *et al.*, 2018); in addition, studies in endometriosis ought to adhere to the WERF EPhect (World Endometriosis Research Foundation Endometriosis Phenome and Biobanking Harmonisation Project) protocols when collecting samples and data (Becker et al., 2014; Fassbender et al., 2014; Rahmioglu et al., 2014; Vitonis et al., 2014).

## Conclusion

Endometriosis is a chronic inflammatory disease with a significant impact on women, their families, and society. The lack of clinically usable biomarkers remains one of the obstacles to shortening waiting times between the first symptoms and endometriosis diagnosis. Currently available medical therapies are mainly aimed at hormonal suppression and disregard the heterogeneity of the biological phenotype and clinical need in women suffering from pain and infertility. sEV-miRNAs could be a promising tool to better understand endometriosis pathophysiology or a potential diagnostic and therapeutic tool. Unfortunately, research in this area is hindered by a lack of standardisation in separating and characterising sEVs and adherence to the WERF EPhect protocols.

## Data Availability

There are no new data associated with this article.
